# Selective Determinants of Low Bone Mineral Mass in Adult Women with Anorexia Nervosa

**DOI:** 10.1155/2013/897193

**Published:** 2013-03-24

**Authors:** Andrea Trombetti, Laura Richert, François R. Herrmann, Thierry Chevalley, Jean-Daniel Graf, René Rizzoli

**Affiliations:** ^1^Bone Diseases Service, Department of Internal Medicine Specialties, Geneva University Hospitals and Faculty of Medicine, Rue Gabrielle-Perret-Gentil 4, 1211 Geneva 14, Switzerland; ^2^Central Laboratory of Clinical Chemistry, Geneva University Hospitals, 1221 Geneva, Switzerland

## Abstract

We investigated the relative effect of amenorrhea and insulin-like growth factor-I (sIGF-I) levels on cancellous and cortical bone density and size. We investigated 66 adult women with anorexia nervosa. Lumbar spine and proximal femur bone mineral density was measured by DXA. We calculated bone mineral apparent density. Structural geometry of the spine and the hip was determined from DXA images. Weight and BMI, but not height, as well as bone mineral content and density, but not area and geometry parameters, were lower in patients with anorexia nervosa as compared with the control group. Amenorrhea, disease duration, and sIGF-I were significantly associated with lumbar spine and proximal femur BMD. In a multiple regression model, we found that sIGF-I was the only significant independent predictor of proximal femur BMD, while duration of amenorrhea was the only factor associated with lumbar spine BMD. Finally, femoral neck bone mineral apparent density, but not hip geometry variables, was correlated with sIGF-I. In anorexia nervosa, spine BMD was related to hypogonadism, whereas sIGF-I predicted proximal femur BMD. The site-specific effect of sIGF-I could be related to reduced volumetric BMD rather than to modified hip geometry.

## 1. Introduction

Significant bone loss is a major feature of anorexia nervosa [1, 2]. Bone mineral density (BMD) has been reported to be reduced by more than 2.5 SD at either the hip or spine in 38% of women with anorexia nervosa and by more than 1.0 SD in 92% [3]. Bone loss predisposes to an increased risk of fractures [2, 4, 5]. The possible mechanisms of bone loss include nutritionally mediated changes in gonadal steroid concentrations [6] as well as alterations in the GH-IGF-I system, with markedly low serum insulin-like growth factor-I (sIGF-I) concentrations [7–11]. IGF-I stimulates bone formation through its interaction with the type I IGF-I receptor on the osteoblast [12–16]. A number of studies have shown an imbalanced state of bone turnover in such patients, with decreased markers of bone formation and increased or normal markers of bone resorption. The latter observation appears to be primarily driven by estrogen deficiency, while decreased bone formation has been attributed to low sIGF-I levels [7, 17]. Indeed, several studies of anorexia patients found lower levels of sIGF-I and a direct correlation of this hormone with weight or body mass index (BMI). However, data demonstrating that reduced serum concentrations of sIGF-I contribute to low bone mineral density and whether different skeletal sites are affected in a similar way are scarce. The relative impact of estrogen deficiency versus low sIGF-I on volumetric density or size is not well established. In a cohort of patients with anorexia nervosa, we studied skeletal site specificity of the association between bone density or size, and sIGF-I level or sex hormone deficiency. 

## 2. Subjects and Methods

### 2.1. Patients and Controls

The study is a retrospective analysis in a selected group of patients with anorexia nervosa which were consecutively referred to our consultation with data prospectively collected. The participants with anorexia nervosa met at a given time DSM-IV criteria [18], including weight less than 85% of expected, intense fear of being fat, disturbed body image, and amenorrhea for three or more consecutive menstrual cycles. Women who had regular uterine withdrawal bleeding while receiving estrogen therapy and women with patterns of concomitant binge eating and purging type were also included. We also included 11 patients who had resumed menses, but still with an active disease. All except one patients had secondary amenorrhea. One patient had primary amenorrhea.

Height was determined by a Holtain stadiometer (Holtain, Crosswell, Wales). Weight was determined on a calibrated scale to the nearest 0.1 kg. Percentage of ideal body weight [18] and body mass index (with weight and height measured in kilograms/height in meters^2^) were calculated. Age at menarche, time since last menstrual period for patients with amenorrhea at time of evaluation, and total duration of amenorrhea were determined, as well as previous and current estrogen use and fracture history during the course of anorexia nervosa. Calcium and protein intakes were recorded by a validated food frequency questionnaire [19] in a subset of 56 patients. Fifty-two subjects completed a validated questionnaire for a detailed assessment of their specific weekly sports activities, including physical education classes, organized sports, recreational activities, walking, and cycling [20]. Their physical activities were computed in hours per week.

Sixty-six normal women of the same age were used as a control population. Controls were recruited mainly among the hospital staff and their families.

### 2.2. Areal Bone Mineral Density (BMD)

Areal BMD of the lumbar spine (LS) in anteroposterior view, femoral neck (FN) and proximal femur (PF) were measured by dual-energy X-ray absorptiometry (DXA) using Hologic QDR 2000 or 4500 instruments (Hologic, Inc., Waltham, MA).

The coefficient of variation of repeated BMD measurements was 1%-2% [21, 22]. The BMD (in g/cm^2^) of the LS (L2–L4), FN, and PF was also expressed as a T-Score which compares individual BMD values to those of a young normal population of the same gender (number of standard deviations from mean) [23]. According to criteria defined by the World Health Organization, T-Scores between –1 and –2.49 indicate osteopenia, whereas T-Scores below −2.5 standard deviations (≤−2.5) indicate osteoporosis [24]. The T-Score was applied in patients older than 18. BMD (g/cm^2^) of the LS, and those of FN were also expressed in terms of standard deviations from mean gender- and age-matched controls (Z-Scores).

Skeletal size was estimated from the scanned bone projected area of LS and PF. At the spine level, the height and the width at three levels (high, middle, and lower level) of vertebral body L2 were measured. Structural geometry parameters of the hip were measured from conventional DXA outputs of the femoral neck region of interest. We measured the internal and external cortical thickness, the diameter of the femoral shaft at a fixed level of the femoral diaphysis (10 lines from the lower edge of the trochanter minor), neck width (area divided by the mean diameter of the femoral neck), and hip length (measuring the central line from the femoral head to the trochanteric region). The number of measured pixels was multiplied by a conversion factor of 1 pixel = 0.43 mm for the Hologic 2000 device and 1 pixel = 0.45 mm for the Hologic 4500 device. We determined the reproducibility of the bone geometry parameters measurements. All measures were done by the same trained technician. Sample size for the studied group was estimated based on Walter et al.'s approximation method [25]. Assuming a minimal intraclass correlation coefficient (ICC) of 0.5 (p0) against a desired of 0.8 (p1), based on *α* = 0.05 and *β* = 0.20, at least 14 participants were required, if measured 3 times. CV (%) was computed as the 100 times the standard deviation divided by the average of 3 measures repeated in 14 patients. The mean CV of the morphological DXA parameters ranged from 0.12% ± 0.06% to 1.09% ± 0.74%. Bone mineral apparent density (BMAD) was calculated as previously described for LS and FN [26, 27]. At the lumbar spine and femoral neck level, BMAD was computed by using the equation BMAD = BMC/projected area^1.5^ and BMAD = BMC/projected area^2^, respectively. 

### 2.3. Serum and Urinary Measurements

Prolactin, FSH, and TSH were determined using standard methods, for patients with amenorrhea and not receiving any form of estrogen. PTH was determined by immunoradiometric assay with an intrassay coefficient of variation of 1.8%–3.4% (Nichols Institute diagnostics). Calcifediol (25-OH-D_3_) was determined by chemiluminescence assay (Nichols Advantage, San Clemente, CA). The first 19 sIGF-I determinations, that is, before March 2002, were performed by RIA after acid-ethanol extraction (Nichols Institute diagnostics). Thereafter, a fully automated chemiluminescence assay (Nichols Institute diagnostics) was used. Intra- and interassay coefficients of variation were 6.4% and 10% for RIA measurement, and 4.8% and 6.7% for chemiluminescence assay. The two assays were highly correlated (*r* = 0.99), with a regression analysis giving the following results in our laboratory: Nichols Advantage IGF-I assay (y) versus Nichols IGF-I by extraction (RIA) assay (*x*);  *y* = 0.84∗*x* − 2.15. RIA values were transformed accordingly.

IGFBP-3 was measured by an enzyme-labeled chemiluminescent immunometric assay (Immulite, DPC). Plasma and urinary calcium, phosphate, and creatinine were measured using an automatic analyser. Plasma calcium was adjusted for protein levels (adjusted Ca = Ca/[(protein/160) + 0.55]). The following parameters were calculated: (a) fasting urinary Ca-to-creatinine ratio was taken as a reflection of net bone resorption (Bone Resorption Index, BRI); (b) renal tubular reabsorption of phosphate (TmPi/GFR) and of calcium (TRCaI) was calculated as described elsewhere [28, 29].

The marker of bone formation osteocalcin was determined with an immunoradiometric assay (CIS-Bio, Gif-sur-Yvette, France). The specific urine marker of bone resorption deoxypyridinoline was measured by fluorescence emission after acid hydrolysis and high-performance liquid chromatography separation (Bio-Rad system, Munich, Germany) and expressed as a creatinine ratio. 

### 2.4. Statistics

Summary results are presented as the mean ± standard deviation. When the data are not normally distributed, the summary descriptive statistics are the median and interquartile range. All non-Gaussian variables were successfully normalized using simple mathematical transformations. Normality was verified by using Shapiro-Francia tests. Comparisons between patients and age-matched controls for continuous variables were performed with a Wilcoxon matched-pairs signed-ranks test to take into account the matched design. Comparisons between two or more groups for continuous variables were performed with Student's *t*-test or ANOVA for unmatched groups. When appropriate, a non-parametric Kruskal-Wallis test was applied. Chi-squared tests were used for comparing proportions. BMI was calculated in kilograms/meters^2^. 

Correlations between bone mineral density and recorded variables were determined by simple or multiple (backward stepwise) linear regression analyses. Standard multiple regression models were constructed for each skeletal site by using duration of anorexia nervosa, total duration of amenorrhea, age at the time of evaluation, BMI, and sIGF-I as covariates. Adjusted regression coefficients and confidence intervals were determined for each covariate. The significance level for two-sided *P* values was 0.05 in all tests. The data were analyzed using the STATA statistical software package (version 9.2; Stata Corporation, College Station, TX).

## 3. Results

### 3.1. Patients' Characteristics

The subjects were in the early twenties (the age range and median age and interquartile range were 15 to 46 years, median: 20, and interquartile range: 16–33), had a low weight (weight range: 26 to 60 kg, median: 43, and interquartile range: 30–55), and a low BMD (Table 1). The median height was 165, with a range of 146 to 180 cm. Their mean T-Scores (in patients aged more than 18 years) were −1.52 ± 1.23 SD for the anterior-posterior view of LS, −0.93 ± 0.92 SD for the FN, and −1.34 ± 0.92 for the PF, significantly lower as compared with the normal population (Table 1). Osteopenia was found in at least one skeletal site in 63% of patients and osteoporosis in 17% of patients. Five patients had a family history of osteoporosis. No differences in BMD expressed as T-Scores were observed between patients with anorexia nervosa alone and those with the binge-purge subtype of anorexia nervosa at any site measured (data not shown). Eight patients reported a history of low trauma fracture (elbow, wrist or hand (*n* = 4), foot or ankle (*n* = 2), rib (*n* = 1), vertebrae (*n* = 1)). Median time to the first fracture was 6 years (IQR: 5) after the onset of the disease. Their BMD tended to be lower, but not significantly, than that observed in patients (*n* = 54) without a history of low traumatic fracture (data not shown). The age of the fractured and the non-fractured was 24.5 ± 7.7 versus 22.1 ± 6.4 years (ns), duration of amenorrhea was 50.4 ± 60.5 versus 21.4 ± 31.0 months (*P* < 0.14), and disease evolution was 7.3 ± 4.5 versus 4.6 ± 4.5 years (ns). Fifty-five patients reported at least one hospitalization during the course of the disease.

Tanner stages at the time of anorexia onset were not recorded. Girls usually complete puberty by ages 15–17, and menarche occurs generally at Tanner stage 4, at the age of 13. In order to clarify this aspect, we determined the number of patients with a disease onset before menarche. Six patients had a disease onset before the first periods. For 3 of them, the interval was lower than 1 year. Three others had a delayed menarche (after 16), 4 to 5 year after disease onset, and thus possibly before pubertal skeletal growth.

Twenty patients (30%) were current estrogen users (median duration: 27 months, IQR: 61). Six patients (9%) were previous estrogen users (median duration: 8 months, IQR: 29). Age, age at menarche, age at the onset of anorexia nervosa, total duration of amenorrhea, protein and calcium intake, sIGF-I, and physical activity did not differ according to estrogen use and menstrual history at the time of examination (Table 2). Amenorrheic women had a significantly lower BMI (15.3 ± 2.2 in untreated women versus 15.6 ± 2.0 in current estrogen users, *P* < 0.05). Age- and sex-adjusted BMD values (Z-Score) were similar (data not shown).

Serum levels of albumin and protein were lower than the normal range in 17% and 29% of patients, respectively (Table 2), while sIGF-I was below the lower limit of the normal level in 58% of patients. The sIGF-I levels correlated with BMI (*r* = 0.43, *P* < 0.001). This was not the case for serum albumin or protein. Eleven patients displayed vitamin D insufficiency (below 50 nmol/L) and one displayed a deficiency (below 25 nmol/L). Among them, six patients had secondary hyperparathyroidism. Mean serum LH and FSH were at the lowest limit of the normal range.

Markers of bone turnover (total alkaline phosphatase, but not osteocalcin, and fasting urinary calcium-to-creatinine ratio) were lower in estrogen users or estrogen replete patients (Table 2). 

### 3.2. Bone Geometry in Patients and Controls (Table 3)

Bone mineral content was lower in patients as compared with controls. Bone area was in contrast similar or tended to be higher in anorexia nervosa patients. Thus, compatible with a reduced volumetric bone density, rather than altered bone geometry, BMAD was lower, as was also cortical thickness at the level of the femoral shaft.

Bone geometry parameters were compared to the control population according to the disease onset, that is, in those below or above the median age of disease onset, and this did not affect the results (data not shown). 

### 3.3. Relationship between BMD, Bone Geometry and Clinical and Biological Variables (Table 4)

LS BMD correlated to the duration of anorexia nervosa, total duration of amenorrhea, sIGF-I, and BMI. FN BMD was associated (*P* < 0.05 for all comparisons, Table 4) with age at the time of evaluation, duration of anorexia nervosa, total duration of amenorrhea, sIGF-I, and IGFBP-3. BMD at any site was not correlated with the degree of physical activity.

To assess the relative contribution of the variables in determining BMD, multiple regression models were constructed using a backward stepwise process. The following parameters were included: duration of anorexia nervosa, total duration of amenorrhea, age at the time of evaluation, BMI, and sIGF-I. Physical activity was not included in the model, as severely affected patients were unable to exert a high level of such activity. Furthermore, many patients were advised to avoid sports activities in order to conserve their energy. Given the high variability of the recorded calcium and protein intakes and the absence of any correlation in the univariate model, these two variables were not included. LS BMD was significantly correlated with total duration of amenorrhea (*P* < 0.01). The serum level of IGF-I was the only significant independent predictor of FN BMD and remained significant even after adjusting for height. We performed the same analysis excluding the 20 patients receiving a hormone replacement therapy at the time of evaluation and found similar results (data not shown).

We examined whether the influence of the sIGF-I level on BMD could be explained by an effect on true volumetric density or on bone size. The sIGF-I levels correlated with FN BMAD (multiple regression model: *r* = 0.001, *P* < 0.04), but not with hip geometry variables (neck width, hip axis length, cortical thickness, and femoral shaft diameter). 

### 3.4. Relationship between Bone Markers and Demographic, Nutritional, or Hormonal Parameters (Table 5)

There was a correlation between osteocalcin (a bone-specific formation marker) and nutritional variables such as BMI, 25-hydroxy vitamin D, sIGF-I, and IGF-BP3. In a multiple stepwise backward regression model, BMI and 25-hydroxy vitamin D were independent predictors of serum osteocalcin levels.

Urinary deoxypyridinoline and calcium-to-creatinine ratios were significantly higher in patients with amenorrhea at the time of evaluation as compared with those spontaneously menstruating or being treated with estrogen (Table 2).

Deoxypyridinoline positively correlated with age at the time of evaluation, amenorrhea at the time of evaluation, 25-hydroxy vitamin D, and PTH levels. In a stepwise multiple regression, the serum PTH level and amenorrhea at the time of evaluation were the most significant predictors of deoxypyridinoline levels, explaining 36% of its variance.

## 4. Discussion

Our study showed a high prevalence and profound degree of low bone mineral mass in women with anorexia nervosa. Indeed, osteoporosis was present in 17% of patients and osteopenia in 63%. Altogether, 12% reported a history of low trauma fracture. Similar figures have been found in other studies [1–3]. Our results suggest that low bone mass is the consequence of a reduced bone density rather than a reduced bone size.

The pathogenesis of bone loss in anorexia nervosa is still not completely understood. It may result from a number of mechanisms, including estrogen deficiency and inadequate nutritional intakes. We found, as other groups, that age-adjusted bone mineral density correlates with the duration of amenorrhea [6, 9, 30]. In our population, estrogen deficiency seemed to equally affect the spine and the proximal femur. Decreased calcium intake and excessive physical activity may also impact on the degree of bone loss, but we did not find any correlation between physical activity and LS BMD. A beneficial effect of physical activity had been suggested in an observational study published by Seeman et al. [31]. Conversely, another recent study showed that excessive moderate loading exercise may put patients at higher risk of low bone mass, but high bone loading activities may provoke bone accrual during recovery [32]. In our study, data about nutritional intakes and exercise were obtained through a questionnaire completed by the patients, with its associated limitations. Since denial is common in anorexia nervosa, these data should be considered with caution. These young women typically over reported food intakes and under reported exercise [33]. We therefore excluded these variables from the multivariate models. Undernutrition and nutritionally dependent factors are likely to play a major role in the bone loss associated with anorexia nervosa. There were marked effects of nutritional status on bone formation markers which correlated with BMI, 25-hydroxy vitamin D, serum IGF-I, and IGFBP-3, in our study as in others [7, 8, 34]. Caloric and protein deprivation in anorexia nervosa leads to increased GH and decreased IGF-I. This could be explained by a downregulation of the GH receptor or its postreceptor mechanisms, inducing a fall in the GH-dependent serum proteins IGF-I and IGFBP-3 levels [35]. The rise in GH is presumably due to decreased feedback inhibition by sIGF-I. Fibroblast growth factor-21 may be a putative factor of GH resistance in anorexia nervosa [36]. 

Our study confirms the low sIGF-I levels previously reported in low-weight patients with anorexia nervosa [9, 10]. Sixty percent of the patients had sIGF-I values below the age-adjusted reference range; yet, only 17% of the patients showed lower than normal serum albumin concentration, a usual indicator of nutritional status. The reason of these discrepancies in the results is not clear. sIGF-I is closely related to nutritional status as suggested by the highly significant correlation with BMI (*P* < 0.001). Several studies showed that sIGF-I concentration is a more reliable index of nutritional status as compared with others markers [37–39].

The most intriguing finding was the high and independent association of sIGF-I and IGFBP-3 levels with age-adjusted FN BMD. We examined whether differences in bone size may explain the association. In a first step, we incorporated body height in the multiple regression model. This adjustment did not affect the relation between FN BMD and sIGF-I. In a second step, parameters of structural geometry of the hip were measured using conventional DXA outputs obtained from the FN region of interest. We measured medial and lateral cortical thickness at a fixed level of the femoral diaphysis, neck width, hip lengths, and femoral shaft diameter. None of them showed any relation with sIGF-I. In a third step, we examined the impact of another analytic strategy, that of BMAD, a volumetric bone density estimate. We found a positive correlation which suggests that the relation between sIGF-I and FN BMD might not be related to differences in bone size. Note that part of the femoral neck region of interest is intracapsular and thus devoid of periosteum. Data on structural geometry of the hip are scarce. A recent study (Hip Structural Analysis) found an increase in the outer and inner diameter of the hip and a decrease in the cortical thickness at the femoral shaft. These structural modifications were not correlated with sIGF-I [40].

Anorexia nervosa affects patients at a time of acquisition of peak bone mass, and one possible consequence is growth retardation and an effect on bone modelling. Short stature may be present in those starting in the infancy or early childhood [41]. For those beginning their eating disorders later, that is, during or after puberty, a study found that majority of patients with anorexia reached their expected height [42]. In our population, the median age of onset of anorexia was 16 years. A small number of patient had a disease onset before menarche, and had probably reached their adult stature and peak bone mass. This may explain the absence of decrease in bone size.

This study has several limitations, particularly the relative heterogeneity of the study population, with three groups of patients at different stages of severity (active disease, and spontaneously menstruated) and taking or not estrogens. It may be advocated that the inclusion of patients who were taking estrogen confounds the interpretation of the results that spine bone mineral density was related to hypogonadism. Although the benefits of estrogen in the bone density issue of patient with anorexia nervosa were refuted in the last decade, a recent publication suggested that physiologic estrogen replacement given transdermally may be beneficial to bone [43]. Excluding those patients taking estrogen, we found similar results. 

Male patients were not included in the study even if they display similar hormonal issues, given the aim of study in analyzing bone size.

In conclusion, in a large cohort of patients with anorexia nervosa, spine and hip BMD values were related to hypogonadism, whereas sIGF-I was the most significant predictor of bone mass at the FN level. Our morphometric analysis suggests that the site-specific effect of sIGF-I could be related to reduced volumetric BMD rather than to modified hip geometry.

## Figures and Tables

**Table 1:** Patients' characteristics: demographic, anthropometric, dietary, and bone data^#^.

Demographic and dietary data	Patients	Controls	*P*-value
Number	66	66	
Age (yrs)	20.0 (16–33)	21 (16–22)	ns
Restricting type (%)	68%		
Age at menarche (yrs)	13.0 (11–16)	13 (11–15)	ns
Age of onset of anorexia (yrs)	16.0 (13–27)		
Duration of illness (yrs)	3.0 (0.7–12)		
History of low traumatic fractures, % (*n*)	12% (8)		
Time since last menstrual period (mo)°	9.0 (3–33)		
Total duration of amenorrhea (mo)	12.0 (2–84)		
Estrogen use, % (*n*)*	39% (26)		
Estrogen use (mo)	38.8 ± 40.3		
Current estrogen use	30% (20)		
Weight (kg)	42.1 ± 6.9	58.9 ± 8.1	<0.007
Weight (kg) before the onset of illness**	54.7 ± 7.5		
Height (cm)	165.0 ± 7.1	164.3 ± 6.0	ns
Body Mass Index (kg/m^2^)	15.4 ± 2.1	21.8 ± 3.0	<0.007
% of ideal body weight	71.1 ± 10.0		
Calcium intake (mg/d)	835 (281–2000)	835 (510–1074)	ns
Protein intake (g/d)	45 (12–132)	42 (25–57)	ns
Protein intake (g/kg of body weight)	1.11 (0.29–2.92)	0.7 (0.4–1.1)	ns
Physical activity (h/week)	5.6 ± 4.0		
Anteroposterior spine BMD (g/cm^2^)	0.887 ± 0.12	1.030 ± 0.12	<0.0001
Femoral neck BMD (g/cm^2^)	0.753 ± 0.11	0.862 ± 0.11	<0.0001
Total hip BMD (g/cm^2^)	0.788 ± 0.11	0.873 ± 0.15	<0.0009

^#^For skew data, median and interquartile (IQR) range are reported. Otherwise, mean ± SD.

°For amenorrheic women at time of examination.

*At any time since the onset of anorexia.

**Applicable if the onset of the disease occurred after the time of menarche.

**Table 2:** Serum and urinary measurements (mean ± SD, or median values). Patient's characteristics by estrogen use and menstrual history.

Laboratory test	Normal range	*N*	All patients	Amenorrheic women^1^	Current estrogen use	Eugonadal women
			*N* = 66	*N* = 38	*N* = 17	*N* = 11
Serum corrected calcium	[2.25–2.60 mmol/L]	64	2.35 ± 0 .09	2.34 ± 0.1	2.35 ± 0.1	2.36 ± 0.1
Serum phosphate	[0.80–1.4 mmol/L]	63	1.3 (0.3)	1.3 (0.2)	1.3 (0.2)	1.5 (0.4)
Serum creatinine	[35–88 *µ*mol/L]	65	71.85 ± 11.32	71.9 ± 12.9	74.1 ± 8.4	67.8 ± 8.6
Serum protein	[61–79 g/L]	45	66.0 ± 6.4	65.9 ± 6.6	65.8 ± 6.4	66.4 ± 6.8
Serum albumin	[35–48 g/L]	64	38.26 ± 4.51	40.5 ± 4.5°	37.3 ± 3.6	38.1 ± 4.7
Serum osteocalcin	[8.8–29.7 *μ*g/L]	44	20.49 ± 11.30	19.2 ± 11.2	19.3 ± 9.0	28.4 ± 14.1
Serum total alkaline phosphatase	[30–125 IU/L]	61	46 (18)	48 (21)	41 (10)*	51 (14)
Serum IGF-I	[116–447 ng/mL]	66	118 (86)	103 (81)	129 (81)	144 (133)
Serum IGFBP-3	[3.3–6.7 *µ*g/mL]	34	4.63 ± 1.21	4.4 ± 1.3	4.9 ± 1.0	4.9 ± 1.2
Serum parathyroid hormone	[1.1–6.8 pmol/L]	58	3.1 (1.5)	3.0 (1.4)	3.0 (1.8)	3.7 (1.3)
25-hydroxyvitamin D	[25–120 nmol/L]	57	74 (43)	69 (36)	76 (66)	73 (65)
Urinary calcium/creatinine	[0.10–0.50 mmol/mmol]	59	0.37 (0.44)	0.6 (0.6)°	0.3 (0.3)	0.2 (0.3)
Urinary D-pyr/cr^2^	[4.2–18.2 nmol/mmol]	52	17.7 (10.1)	18.1 (7.0)	13.4 (11.0)	16.0 (10.1)
TRCaI/GFR^3^	[2.4–2.9 mmol/L GFR]	55	2.59 ± 0 .21	2.54 ± 0.2	2.64 ± 0.2	2.67 ± 0.2
TmPi/GFR^4^	[0.8–1.4 mmol/L GFR]	55	1.4 (0.3)	1.4 (0.3)	1.3 (0.3)	1.5 (0.5)

Age-adjusted normal values were taken into account.

^1^At the time of evaluation, and including 3 patients taking oral contraceptives, but continuing to be amenorrheic.

^2^Deoxypyridinoline/creatinine.

^3^Renal tubular reabsorption of calcium index.

^4^Renal tubular reabsorption of phosphate.

°*P* < 0.05 versus current estrogen use and eugonadal women.

*P < 0.05 current estrogen versus eugonadal women.

**Table 3:** Bone geometry as measured by DXA in patients with anorexia nervosa and in controls.

	Patients	Controls	*P*
Lumbar spine			
BMC (g)	40.1 ± 7.2	43.6 ± 7.1	0.01
Area (cm^2^)	44.9 ± 4.8	42.3 ± 4.0	0.002
BMAD (g/cm^3^)	0.13 ± 0.01	0.16 ± 0.02	0.0001
L2 vertebral height (mm)	29.1 ± 1.8	28.0 ± 2.3	0.0029
L2 width: higher part (mm)	40.2 ± 2.9	38.9 ± 3.3	0.02
L2 width: middle part (mm)	34.8 ± 2.7	33.8 ± 2.4	ns
L2 width: lower part (mm)	38.5 ± 3.2	35.3 ± 2.9	0.001
Hip			
BMC (g)/neck	4.0 ± 0.7	4.4 ± 0.7	0.003
Area (cm^2^)/neck	5.3 ± 0.4	5.1 ± 0.5	0.01
BMC (g)/total hip	24.1 ± 4.2	29.3 ± 3.9	0.0001
Area (cm^2^)/total hip	31.2 ± 3.1	30.9 ± 2.0	ns
Hip midline (mm)	83.7 ± 4.6	81.0 ± 5.0	0.002
Neck diameter (mm)	31.1 ± 2.2	30.3 ± 2.1	ns
BMAD (g/cm^3^)	0.14 ± 0.02	0.17 ± 0.03	0.0001
BMC (g)/trochanter	5.4 ± 1.1	6.3 ± 1.3	0.0007
Area (cm^2^)/trochanter	9.0 ± 1.0	8.9 ± 0.9	ns
Femoral shaft (mm)	27.2 ± 2.2	26.5 ± 2.5	ns
Internal cortical thickness (mm)	5.8 ± 1.0	6.3 ± 0.9	0.008
External cortical thickness (mm)	5.0 ± 0.8	5.0 ± 0.8	ns

Mean ± SD.

**Table 4:** Relationship of BMD and variables measured (univariate regression analyses and multiple regression models constructed by backward stepwise).

	AP spine BMD						Femoral neck BMD					
Variables	Univariate			Multivariate			Univariate			Multivariate		
	Regres. coeff. (95% CI)	*P*	*R* ^2^	Regres.coeff. (95% CI)	*P*	*R* ^2^	Regres. coeff. (95% CI)	*P*	*R* ^2^	Regres. coeff. (95% CI)	*P*	*R* ^2^
Age (yrs)^#^	0.29 (−0.81; 1.39)	ns					1.24 (0.28; 2.19)	0.01	9%			
Disease duration (yrs)°	−0.01 (−0.02; −0.00)	0.03	7%				−0.01 (−0.02; −0.00)	0.01	10%			
Duration of amenorrhea (months)*	−0.03 (−0.06; −0.01)	0.01	10%	−0.03 (−0.05; −0.01)	0.01		−0.03 (−0.05; −0.00)	0.02	8%			
BMI (Kg/m^2^)	0.02 (0.00; 0.03)	0.02	9%				0.01 (−0.01; 0.02)	ns				
IGF-I (*µ*g/mL)°	0.01 (0.00; 0.02)	0.01	11%				0.02 (0.01; 0.02)	0.001	22%	0.01 (0.01; 0.02)	0.001	
IGFBP-3 (mg/mL)	0.01 (−0.01; 0.04)	ns					0.03 (0.00; 0.05)	0.02	16%			
Parathyroid hormone^#^	0.11 (−0.14; 0.35)	ns					0.19 (−0.05; 0.44)	ns				
25 OH-Vitamin D (nmol/l)°	0.00 (−0.01; 0.02)	ns					−0.001 (−0.01; 0.01)	ns				
Physical activity (h/week)°	0.03 (−0.00; 0.06)	ns					0.01 (−0.02; 0.04)	ns				
Protein intake (g/kg of body weight × d)°	−0.07 (−0.16; 0.01)	ns					−0.04 (−0.11; 0.04)	ns				
Calcium intake (mg/d)*	−0.04 (−0.10; 0.02)	ns					−0.02 (−0.07; 0.03)	ns				
						21%						29%

^#^Reciprocal root variable (1/square root).

°Square root transformed variable.

*Log transformed variable.

**Table 5:** Relationship between bone markers and demographic, nutritional, or hormonal parameters (univariate regression analyses).

Variables	Serum osteocalcin	(*n* = 44)		Urinary deoxypyridinoline	(*n* = 52)	
	Regres. coef. (95% CI)	*P* univar.	*R* ^2^	Regres. coef. (95% CI)	*P* univar.	*R* ^2^
Age (yrs)	−0.114 (−0.600; 0.373)	ns		−0.349 (−0.716; 0.017)	0.06	7%
Disease duration (yrs)	−0.205 (−0.989; 0.580)	ns		−0.520 (−1.049; −0.008)	0.053	5%
Total duration of amenorrhea (months)	0.024 (−0.077; 0.125)	ns		0.000 (−0.078; 0.08)	ns	
BMI (Kg/m^2^)	3.157 (1.416; 4.898)	0.001	24%	−0.450 (−1.561; 0.660)	ns	
IGF-I (*μ*g/mL):	0.040 (−0.001; 0.08)	0.058	8%	−0.002 (−0.031; 0.027)	ns	
IGFBP-3 (mg/mL)	3.76 (0.630; 6.889)	0.021	24%	1.0 (−1.319; 3.314)	ns	
Estrogen use	−2.342 (−10.121; 5.436)	ns		−4.702 (−9.34; −0.059)	0.05	8%
Parathyroid hormone (pmol/L)	0.831 (−3.268; 1.604)	ns		−1.743 (−3.014; −0. 472)	0.01	14%
25-Hydroxyvitamin D (nmol/L)	−0.103 (−0.198; −0.009)	0.03	12%	−0.039 (−0.082; 0.002)	0.063	8%
Protein intake (g/kg of body weight × d)	−1.996 (−5.116; 1.124)	ns		0.027 (−3.080; 3.134)	ns	
Calcium intake (mg/d)	−0.000 (−0.006; 0.006)	ns		0.000 (−0.006; 0.004)	ns	
